# Assessment of left ventricular ejection fraction using an ultrasonic stethoscope in critically ill patients

**DOI:** 10.1186/cc11198

**Published:** 2012-02-15

**Authors:** Jean-Bernard Amiel, Ana Grümann, Gwenaëlle Lhéritier, Marc Clavel, Bruno François, Nicolas Pichon, Anthony Dugard, Benoît Marin, Philippe Vignon

**Affiliations:** 1 Medical-surgical Intensive Care Unit, Dupuytren Teaching Hospital, Avenue Martin Luther King, 87000 Limoges, France; 2 CIC-P 0801, Dupuytren Teaching Hospital, Avenue Martin Luther King, 87000 Limoges, France; 3 Unité Fonctionnelle de Recherche Clinique et Biostatistique, Dupuytren Teaching Hospital, Avenue Martin Luther King, 87000 Limoges, France; 4 University of Limoges, 2 Avenue Dr Marcland, 87000, Limoges, France

## Abstract

**Introduction:**

Assessment of cardiac function is key in the management of intensive care unit (ICU) patients and frequently relies on the use of standard transthoracic echocardiography (TTE). A commercially available new generation ultrasound system with two-dimensional imaging capability, which has roughly the size of a mobile phone, is adequately suited to extend the physical examination. The primary endpoint of this study was to evaluate the additional value of this new miniaturized device used as an ultrasonic stethoscope (US) for the determination of left ventricular (LV) systolic function, when compared to conventional clinical assessment by experienced intensivists. The secondary endpoint was to validate the US against TTE for the semi-quantitative assessment of left ventricular ejection fraction (LVEF) in ICU patients.

**Methods:**

In this single-center prospective descriptive study, LVEF was independently assessed clinically by the attending physician and echocardiographically by two experienced intensivists trained in critical care echocardiography who used the US (size: 135 × 73 × 28 mm; weight: 390 g) and TTE. LVEF was visually estimated semi-quantitatively and classified in one of the following categories: increased (LVEF > 75%), normal (LVEF: 50 to 75%), moderately reduced (LVEF: 30 to 49%), or severely reduced (LVEF < 30%). Biplane LVEF measured using the Simpson's rule on TTE loops by an independent investigator was used as reference.

**Results:**

A total of 94 consecutive patients were studied (age: 60 ± 17 years; simplified acute physiologic score 2: 41 ± 15), 63 being mechanically ventilated and 36 receiving vasopressors and/or inotropes. Diagnostic concordance between the clinically estimated LVEF and biplane LVEF was poor (Kappa: 0.33; 95% CI: 0.16 to 0.49) and only slightly improved by the knowledge of a previously determined LVEF value (Kappa: 0.44; 95% CI: 0.22 to 0.66). In contrast, the diagnostic agreement was good between visually assessed LVEF using the US and TTE (Kappa: 0.75; CI 95%: 0.63 to 0.87) and between LVEF assessed on-line and biplane LVEF, regardless of the system used (Kappa: 0.75; CI 95%: 0.64 to 0.87 and Kappa: 0.70; CI 95%: 0.59 to 0.82, respectively).

**Conclusions:**

In ICU patients, the extension of physical examination using an US improves the ability of trained intensivists to determine LVEF at bedside. With trained operators, the semi-quantitative assessment of LVEF using the US is accurate when compared to standard TTE.

## Introduction

In intensive care unit (ICU) settings, several studies have long shown that physical examination was inaccurate in predicting the hemodynamic status of patients with circulatory or respiratory failure, even when performed by experienced intensivists [1,2]. Specifically, the range of cardiac index (low, normal or high) has been shown to be adequately predicted by the physical examination in only 44 to 51% of ICU patients who were evaluated using right heart catheterization [1-3]. Ejection fraction (EF) is the most commonly used parameter of left ventricular (LV) systolic function on clinical grounds [4]. This parameter is altered not only by LV contractility (and inotropes), but also by LV volumes, preload, afterload (and vasopressors) and valvular function [5]. Nevertheless, LVEF has the advantage of internally normalizing the stroke volume by LV end-diastolic volume and, therefore, can be used as a parameter of LV systolic function that is independent of the size of the patient or the ventricle [6]. Although LVEF fails to directly reflect systolic flow or the overall circulatory state, the determination of cardiac pump function is key in the management of ICU patients with cardiorespiratory compromise.

Standard transthoracic echocardiography (TTE) is currently the first-line imaging modality recommended for the measurement of LVEF [4]. Miniaturized, battery-operated systems have been successfully used in ICU patients [7,8]. The recent emergence of commercially available pocket-size miniaturized ultrasound devices virtually enables physicians to extend the physical examination with an ultrasonic stethoscope (US) [9]. This approach has been validated for the qualitative evaluation of LVEF in cardiology patients [10], but not yet in the ICU settings. Accordingly, the primary endpoint of this study was to evaluate the additional value of an US for the determination of LV systolic function, when compared to conventional clinical assessment by experienced intensivists. The secondary endpoint was to validate the US against TTE for the semi-quantitative assessment of LVEF in ICU patients.

## Materials and methods

### Patients

This single-center prospective descriptive study was approved by our institutional Ethical Committee, which waived the need for informed consent. During a six-week period, all patients hospitalized in our medical-surgical ICU underwent, systematically, a clinical and echocardiographic assessment of LVEF using standard TTE and a new generation US within the first 12 hours of admission. The presence or absence of congestive heart failure was clinically evaluated by the attending physician (senior intensivist with at least five-years' experience in critical care medicine) based on medical history, physical examination and admission chest X-ray as previously described [11], or on any other available information, including previously measured LVEF. In each patient, the clinically or echocardiographycally estimated LVEF was classified in one of the following categories: increased (LVEF > 75%), normal (LVEF: 50 to 75%), moderately reduced (LVEF: 30 to 49%), or severely reduced (LVEF < 30%). Hypotension was defined as a systolic blood pressure < 90 mmHg or a mean blood pressure < 65 mmHg. Shock was defined as the presence of clinical signs of tissue hypoperfusion confirmed by a metabolic acidosis, high lactate level or a decreased ScvO_2 _associated with a sustained hypotension, or not [12]. Patients were not studied when < 18 years or when one of the investigators was not available.

### Echocardiographic assessment of LVEF

**Figure 1:**
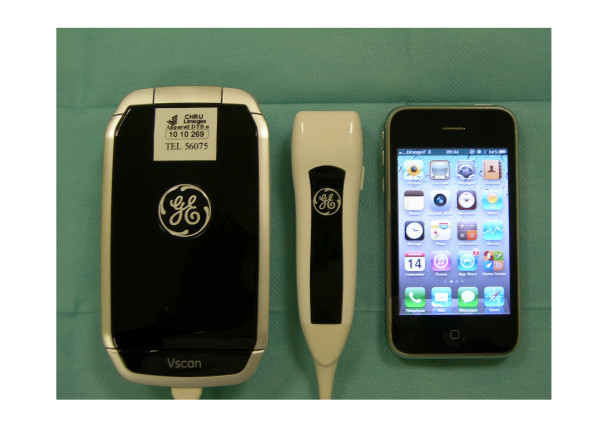
**New generation miniaturized ultrasound system the size of a smartphone used in the current study**.

The tested US was a new generation (VScan™, General Electrics Healthcare) miniaturized (size: 135 × 73 × 28 mm; weight: 390 g), battery-operated (total scan time of one hour) device with a broad-band width (1.7 to 3.8 MHz) phased array probe (120 × 33 × 26 mm). This system can store digital still-frames or image loops, uses a color-coded overlay for real-time blood flow imaging, and allows distance measurements using integrated electronic calipers, but has neither spectral nor tissue Doppler capability (Figure 1). TTE studies were performed with a full-feature system (CX50, Philips Healthcare).

The two echocardiographic examinations were performed independently, and randomly according to the availability of investigators and ultrasound systems, but within the same hour. Operators were experienced intensivists highly trained in critical care echocardiography [13]. The following TTE views were systematically screened in each patient: parasternal short-axis view, apical four- and two-chamber views and subcostal four-chamber view. In each TTE view, imaging quality was graded as 0 (no image), 1 (less than 50 percent of endocardial border well delineated), 2 (more than 50 percent of endocardial border well delineated) and 3 (entire endocardial border well delineated) [7]. An overall quality score was calculated by adding imaging quality grades of all four studied TTE views (range: 0 to 12). Three digital loops were recorded during TTE at end-expiration in the apical four- and two-chamber views for further analysis. The time required to perform the examination purposely focused on the visual estimation of LVEF (from the first image obtained to the end of examination) was recorded.

The investigators assessed semi-quantitatively LVEF using TTE or the US in the apical four- and two-chamber views, with the same four-subset classification than that used for the clinical evaluation. The echocardiographers and the front-line intensivists who clinically assessed LVEF had access to the same information but were not allowed to communicate until individual clinical research forms had been independently fulfilled at the patient's bedside. LVEF was then measured off-line by two independent intensivists with an ASE level 3 competence in echocardiography [14]. LV end-diastolic volume (LVEDV) and LV end-systolic volume (LVESV) were measured in the apical four- and two-chamber views using the Simpson's method, according to current recommendations [15]. LV volumes were measured on three non-consecutive cardiac cycles and averaged. LVEF was conventionally calculated as the stroke volume (LVEDV-LVESV) normalized by LVEDV [5]. Values derived from both the apical four- and two-chamber views were then averaged to obtain individual biplane LVEF, unless image quality of the apical two-chamber view was not suitable for LV volume measurements. TTE-derived LVEF values were used to determine the reference category of individual systolic cardiac function.

### Statistical analysis

Results are expressed as means ± standard deviations or frequencies and percentages. Paired comparisons were performed using the paired Student *t-*test for continuous variables and with the Mac Nemar Chi-2 test for qualitative variables. Percent comparisons were performed using Pearson's Chi-2 test. Interobserver variability of LVEF measurement was computed as the absolute difference between the two sets of measurements divided by the mean of the two values and expressed as a percentage of error. Diagnostic concordance for the semi-quantitative evaluation of LVEF at patient's bedside was assessed using a weighted Kappa test with 95% confidence intervals (CI) as follows: clinical evaluation vs. measured LVEF, visual assessment using the US and TTE vs. measured LVEF, and visual assessment using the US vs. visual assessment using TTE. A *P-*value < 0.05 was considered statistically significant.

## Results

Among the 122 patients admitted to our ICU during the study period, 33 were excluded due to the unavailability of one investigator (*n *= 26), or an age < 18 years (*n *= 2). Finally, 94 patients were studied (mean age: 60 ± 17 years; men: 71; mean simplified acute physiologic score (SAPS 2): 41 ± 15). Reasons for admission to the ICU were predominantly a circulatory failure (*n *= 56) or an acute respiratory failure (*n *= 26). Sixty-three patients (67%) were mechanically ventilated and 36 patients (38%) received vasopressors and/or inotropes (Table 1). Mean duration of echocardiographic examination was significatively shorter when using the US (241 ± 107 vs. 300 ± 131 s: *P *= 0.0014) but its overall mean image quality was inferior to that of TTE (5.9 ± 3.7 vs 6.9 ± 3.7: *P *= 0.0009). Noticeably, the proportion of good-to-excellent image quality grade in the apical four-chamber view used to visually assess LVEF was not significantly different with the US and TTE (grades 2 and 3: 54/94 vs 61/94; *P *= 0.37), and LVEF was not visually evaluable in a similar proportion of patients (12/94 vs 10/94: *P *= 0.50).

**Table 1:** Characteristics of the study population

	Descriptive parameters
Age, yr	60 ± 17
Men, n	71 (74%)
SAPS II	41 ± 15
Invasive mechanical ventilation, n	63 (66%)
Reason for admission, n*:	
shock	35
hypotension	16
cardiac arrest	5
acute respiratory failure	26
systematic assessment	28
Hemodynamic management*:	
fluid loading, mL	478 ± 967
vasopressor support, n	26 (28%)
inotropes, n	10 (11%)
Blood pressure, mmHg:	
systolic	120 ± 27
diastolic	68 ± 15
mean	84 ± 19
Heart rate, bpm	91 ± 24
Central venous pressure, mmHg	9 ± 4
V_T_, mL	460 ± 63
Plateau pressure, cmH_2_O	19 ± 4
PEEP, cm H_2_O	7 ± 3
PaO_2_/FiO_2_	251 ± 112
Lactates, mmol/L	4.04 ± 3.92
Creatinin, μmol/L	143 ± 158
ALAT, IU/L	93 ± 196
Hemoglobin, g/dL	9 ± 2

*: a single patient may fulfill several criteria

LVEF was measured off-line in 84 patients (89%) in the apical four-chamber view and in 44 patients (47%) in the apical two-chamber view. Interobserver variability for LVEF measurement was 9 ± 7%. Mean LVEF was 54 ± 19%. According to TTE-derived LVEF values, 8 patients had an increased LVEF, 51 patients had a normal LVEF, 14 patients had a moderately reduced LVEF and the remaining 11 patients had a severely reduced LVEF. Diagnostic concordance between the clinically estimated LVEF by the attending physicians and the measured LVEF was poor (Kappa: 0.33; 95% CI: 0.16 to 0.49), and only slightly improved by the knowledge of a previously determined LVEF value (*n *= 21; Kappa: 0.44; 95% CI: 0.22 to 0.66). The sensitivity and specificity of the clinical judgement to identify a decreased LVEF (that is, < 50%) was 60% (95% CI: 39 to 79) and 83% (95% CI: 71 to 92), respectively.

In contrast, the diagnostic agreement was good between the visually assessed LVEF using the US and TTE (Kappa: 0.75; CI 95%: 0.63 to 0.87). Similarly, the visually assessed LVEF using the US and TTE was in agreement with the measured reference values (Kappa: 0.75; 95% CI: 0.64 to 0.87 and Kappa: 0.70; 95% CI: 0.59 to 0.82, respectively). When compared with the reference LVEF, the visual assessment using the US overestimated LVEF in nine patients and underestimated LV systolic function in a single patient (Table 2). Similarly, TTE led to visually overestimate LVEF in 12 patients and to underestimate LV systolic function in 8 patients when compared to reference measurements (Table 3). When the reference LVEF was in the normal or increased range of values, LV systolic dysfunction was erroneously identified in only three patients. Regardless of the ultrasound system used, most inaccurate visual assessments of LVEF were related to an inadequate evaluation of the severity of LV systolic dysfunction or to the erroneous distinction between a normokinetic and a hyperkinetic LV (Tables 2 and 3).

**Table 2:** Visual assessment of left ventricular ejection fraction (LVEF) using the ultrasound stethoscope*

	Visual assessment of LVEF with the ultrasound stethoscope			
**Reference LVEF**	**Increased** **LVEF > 75%**	**Normal** **LVEF: 50 to 75%**	**Moderately depressed** **LVEF: 30 to 49%**	**Severely depressed** **LVEF < 30%**
Increased	4	4	0	0
Normal	1	47	1	0
Moderately depressed	0	3	11	0

Severely depressed	0	1	4	6

*: Biplane LVEF measured off-line with the Simpson's rule using an upper-end system was used as a reference.

**Table 3:** Visual assessment of left ventricular ejection fraction (LVEF) using the full-feature ultrasound system*

	Visual assessment of LVEF with the full-feature system			
**Reference LVEF**	**Increased** **LVEF > 75%**	**Normal** **LVEF: 50 to 75%**	**Moderately depressed** **LVEF: 30 to 49%**	**Severely depressed** **LVEF < 30%**
Increased	3	5	0	0
Normal	6	43	2	0
Moderately depressed	0	2	11	1

Severely depressed	0	0	4	7

*: Biplane LVEF measured off-line with the Simpson's rule using the same ultrasound system was used as a reference.

## Discussion

In this study, LVEF could not be accurately predicted in ICU patients by the sole physical examination and the knowledge of a previously determined LVEF value failed to significantly improve the clinical judgement of the front-line intensivist. In contrast, the herein tested new generation US allowed an accurate semi-quantitative assessement of LVEF when compared with standard TTE, during a focused, rapid-to-perform examination.

Previous studies have long shown that physical examination was inaccurate in predicting the hemodynamic status of ICU patients (for example, cardiac index, cardiac filling pressures, systemic vascular resistance) when using right heart catheterization as a reference. In these series, the cardiac index was adequately graded as low, normal or high in only approximately half of the cases when compared to measurements obtained by the thermodilution technique [1,2]. Similarly, front-line intensivists adequately predicted LVEF in only 64 of our patients (68%), as reflected by a poor diagnostic agreement between the clinical assessment and the reference LVEF value. Importantly, the use of an US as an extension of the physical examination markedly improved the clinical evaluation of cardiac function. In a systematic review of the literature, Badgett *et al. *[11] reported that the performance of the physical examination for detecting a decreased LVEF or increased LV filling pressure was fairly poor in ICU patients, with an overall sensitivity and specificity of 54% and 69%, respectively. In the present study, the sensitivity and specificity of the clinical examination to identify a decreased LVEF were slightly higher (60% and 83%, respectively). Interestingly, the knowledge of a previous LVEF value failed to significantly improve the clinical judgement in our patients. This result is presumably related to the frequency of transient LV systolic dysfunction in ICU patients who sustain acute insults [16,17] and to the beneficial effects of ongoing inotropic support which may have variously improved LV systolic function.

As previously reported using the same US [18], a fairly good two-dimensional imaging quality was obtained in most of our ICU patients, of which the majority was mechanically ventilated. Accordingly, the diagnostic concordance between the US and TTE was good for the semi-quantitative assessment of LVEF. This result is in keeping with those of previous studies performed in other medical settings which reported a high concordance for the diagnosis of decreased LVEF using the herein tested US and TTE as a reference [10,19]. Interestingly, the number and nature of LVEF misclassifications were similar using the two TTE approaches in our patients and were predominantly related to the distinction between moderately and severely reduced LVEF, and between normal and hyperkinetic LV wall motion. Other commercially available US appear promising in providing accurate information on cardiac chamber size and function, but have yet been only scarcely tested in cardiology patients [20].

Visual assessment of LV systolic function using TTE has been shown to be reliable when performed by trained operators [21]. In the ICU settings, we [22,23] and others [24] have recently reported that a tailored training program allowed residents without previous experience in ultrasound to accurately assess semi-quantitatively global LV systolic function as normal or increased, reduced or severely reduced. Interestingly, the diagnostic agreement between the trained residents and the experienced intensivists or cardiologists was good to excellent in all these studies (Kappa: 0.76 (CI 95%: 0.59 to 0.93); Kappa: 0.84 (CI 95%: 0.76 to 0.92); Kappa: 0.68 (CI 95%: 0.48 to 0.88)) [22-24]. A recent study performed in ambulatory patients showed that residents of internal medicine who received a 15-hour training program adequately assessed LVEF using the same US than that used in the present study (Kappa: 0.87) [19]. Whether the new generation of US will allow trainees who are novice in ultrasound to be reliable when performing basic level critical care echocardiography remains to be determined [13].

Taken together, these results suggest that the tested US is reliable to semi-quantitatively assess LVEF during a short, focused examination in ICU patients. Importantly, the European Association of Echocardiography recently stated that current pocket-size imaging devices should only be considered as screening tools or used to complement the physical examination in various clinical settings, including in ICUs [25]. The range of indications is limited and the results of US should be reported as part of the physical examination. Finally, a specific training and certification is recommended for all users and the patient has to be informed that pocket-size imaging systems fail to replace standard TTE [25]. In addition to the semi-quantitative evaluation of LVEF, the US appears promising to quickly evaluate in ICU patients the right ventricular size and function, the presence and volume of pericardial and pleural effusions as well as the size and respiratory variations of the inferior vena cava, due to its two-dimensional imaging quality.

The present study has several limitations. Although technically possible, LVEF has not been measured off-line on images obtained with the US. Nevertheless, the concept of US is based on a target-oriented (for example, LVEF assessment), rapid evaluation to obtain information which is not accessible to physical examination. Accordingly, off-line measurement of LV volumes using the specific software provided with US is not clinically relevant. Both the order of echocardiographic examinations and allocation of ultrasound systems were not randomized, but rather depended on the availability of devices and investigators. Nevertheless, this potential methodological bias should have a minor impact on the observed results since surface echocardiography has long been used in our ICU by highly trained operators [26]. Accordingly, the present results cannot be generalized to less experienced operators. Additional information provided by the US was purposely not analyzed, especially that related to the use of color Doppler mapping. Accordingly, the present study failed to validate the tested US to perform basic critical care echocardiography [13]. Finally, the therapeutic impact related to the use of an US as an extension of physical examination in the ICU settings remains to be determined.

## Conclusions

In ICU patients, the extension of physical examination using an US improves the ability of trained intensivists to determine LVEF at the bedside. With trained operators, the semi-quantitative assessment of LVEF using the US is accurate when compared to standard TTE.

## Key messages

• In the present study, the pocket-size device used as an ultrasonic stethoscope (US) by intensivists trained in critical care echocardiography improved the clinical evaluation of left ventricular ejection fraction (LVEF) in intensive care unit (ICU) patients

• In this setting, the tested US was accurate for the semi-quantitative evaluation of LVEF when using standard transthoracic echocardiography (TTE) as a reference

• The concordance of visually estimated LVEF using the US and TTE on the one hand, and the biplane LVEF value on the other hand, were similar in our ICU patients

• These results should not be extrapolated to other indications of echocardiography and to less experienced examiners. The design of the present study does not allow us to validate the use of this US to perform basic critical echocardiography.

## Abbreviations

CI: confidence interval; EF: ejection fraction; ICU: intensive care unit; LV: left ventricle; LVEDV: left ventricular end-diastolic volume; LVESV: left ventricular end-systolic volume; TTE: transthoracic echocardiography; SAPS: simplified acute physiologic score; US: ultrasonic stethoscope

## Competing interests

The authors declare that they have no competing interests.

## Authors' contributions

JBA, GL, AD, MC, BF, NP and PV participated in the acquisition of data. BM, AG and PV participated in the analysis and interpretation of data. JBA and PV contributed to the conception and design of the study and were involved in the drafting and revision of the manuscript. All authors read and approved the version of the manuscript to be submitted.
